# Interaction of hypertension and remnant cholesterol on arterial stiffness in Chinese middle-aged and elderly populations

**DOI:** 10.1186/s12944-026-03006-0

**Published:** 2026-06-30

**Authors:** Fuliang Yi, Xuena Zou, Wenzhu Song, Xin Liu, Yujuan Ying, Qiaojin Xie, Yang Gao

**Affiliations:** 1 Zigong Fourth People’s Hospital, Zigong, Sichuan Province 643000 China; 2 School of Public Health, Zhejiang University, Hangzhou, Zhejiang Province 310030 China; 3 Zigong First People’s Hospital, Zigong, Sichuan Province 643000 China

**Keywords:** Arterial stiffness, Hypertension, Remnant cholesterol, Middle-aged and elderly population, Additive interaction

## Abstract

**Objective:**

Arterial stiffness (AS) is a critical pathological basis for major cardiovascular diseases (CVD), with its progression closely associated with modifiable risk factors such as hypertension and dyslipidemia. Remnant cholesterol (RC), a key dyslipidemia indicator, is increasingly recognized for its cardiovascular risk predictive value independent of low-density lipoprotein cholesterol (LDL-C). This study explored the independent and additive interactive associations of hypertension and RC with AS in a Chinese middle-aged and elderly population, so as to provide evidence-based support for the prevention and management of cardiovascular diseases in this high-risk population.

**Methods:**

A retrospective cohort study was conducted on 6,886 middle-aged and elderly participants (≥ 45 years) who underwent health examinations at the Health Management Center of Zigong Fourth People’s Hospital from January to December 2023. All participants completed measurements of brachial-ankle pulse wave velocity (baPWV) for AS assessment, blood pressure, blood lipids, and other biochemical indicators. Participants were stratified into the AS group (baPWV ≥ 1800 cm/s) and normal group (baPWV < 1800 cm/s). Multiple analytical approaches were used to analyze the relationship between hypertension, RC and AS: multivariable logistic regression models (adjusted for age, gender, BMI, smoking history, drinking history, diabetes, and other confounding factors) were applied to evaluate their independent and joint effcets with AS; participants were further divided into four subgroups (low RC without hypertension, high RC without hypertension, low RC with hypertension, high RC with hypertension) to assess the interaction of the two factors on AS risk; additive interaction models were constructed to quantify their additive interaction; restricted cubic spline (RCS) models were used to explore the non-linear relationship between blood pressure and AS risk and to identify apparent inflection points within the study population.

**Results:**

Of the 6,886 participants, 4,702 (68.3%) were identified with AS. Both hypertension and elevated RC were independently associated with an increased odds of AS (*P* < 0.001). Participants with both hypertension and high RC exhibited the highest risk of AS (OR = 3.50, 95% CI: 2.66–4.61) compared to the low RC without hypertension. A additive interaction between hypertension and elevated RC on AS was observed, with the relative excess risk due to interaction (RERI) = 1.21 (95% CI: 0.13–2.23). The RCS model identified apparent inflection points around 127 mmHg for systolic blood pressure (SBP) and 84 mmHg for diastolic blood pressure (DBP), where the risk of AS appeared to increase more rapidly within the study population.

**Conclusion:**

This study identified an additive interaction between hypertension and elevated RC in relation to AS among middle-aged and elderly Chinese adults. These findings suggest that the integrated assessment of hypertension and RC may provide complementary information for identifying individuals at increased risk of AS.

**Supplementary Information:**

The online version contains supplementary material available at https://doi.org/10.1186/s12944-026-03006-0.

## Introduction

Arterial stiffness (AS), characterized by progressive thickening, hardening, and loss of elasticity of the arterial wall, is a key precursor to cardiovascular diseases (CVD) such as coronary heart disease, ischemic stroke, and peripheral arterial disease [1–3].

As a non-invasive, sensitive and widely recognized indicator for assessing AS, brachial-ankle pulse wave velocity (baPWV) can effectively detect early vascular injury, and its elevation is closely correlated with an elevated risk of adverse cardiovascular events [4]. According to the 2021 Global Burden of Disease Study, AS-related diseases accounted for over 250 million disability-adjusted life years (DALYs) worldwide, with hypertension and dyslipidemia being the most influential modifiable risk factors [5].

Remnant cholesterol (RC), derived from the metabolism of triglyceride-rich lipoproteins (chylomicrons and very-low-density lipoproteins), has emerged as a critical marker of residual cardiovascular risk. Unlike low-density lipoprotein cholesterol (LDL-C), RC can explain the additional cardiovascular risk that persists even after optimal LDL-C control [6]. Middle-aged and elderly populations are particularly vulnerable to AS due to age-related vascular aging, declining metabolic function, and a high comorbidity rate of hypertension and dyslipidemia [7]. Previous studies have demonstrated that remnant cholesterol (RC) is independently associated with AS and cardiovascular risk [8, 9]. In addition, recent evidence has suggested that blood pressure may partially mediate the association between RC and AS among hypertensive individuals [10]. Whether hypertension and elevated RC jointly contribute to AS through an additive interaction remains unclear.

Therefore, this study aimed to investigate the independent and joint effects of hypertension and RC on AS and to quantify their additive interaction in a large Chinese middle-aged and elderly population.

## Participants and methods

### Study participants

**Fig. 1:**
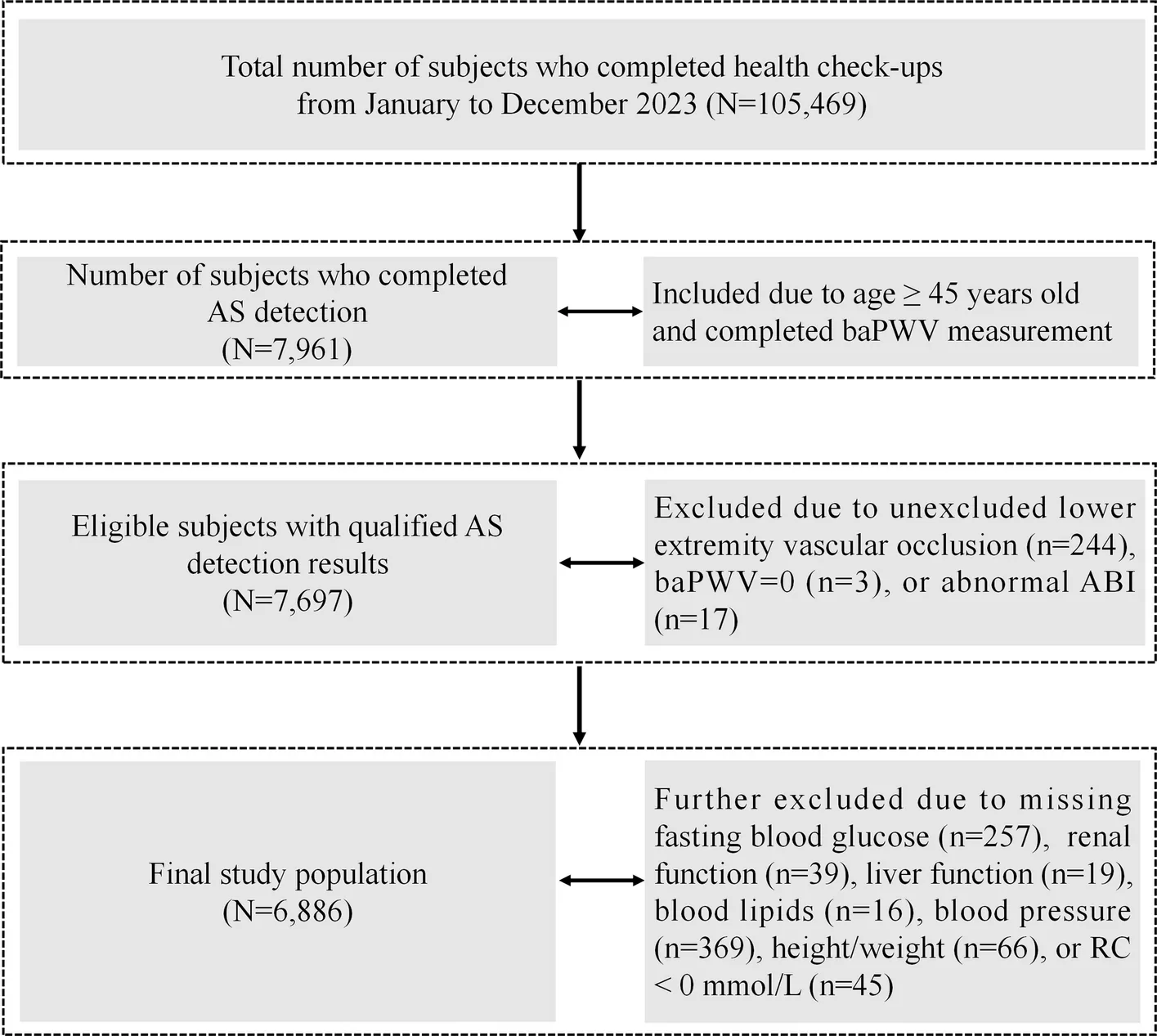
Flowchart of subject inclusion and exclusion criteria

A retrospective analysis was conducted on 6,886 middle-aged and elderly participants who underwent AS assessment at the Health Management Center of Zigong Fourth People’s Hospital from January to December 2023. Participants were enrolled based on the following inclusion and exclusion criteria: Inclusion criteria: (1) Aged ≥ 45 years; (2) Completed baPWV measurement; (3) Completed blood pressure measurement and blood lipid testing (including total cholesterol (TC), triglycerides (TG, high-density lipoprotein cholesterol (HDL-C), LDL-C); (4) Provided complete medical history (smoking, drinking, diabetes, dyslipidemia) and biochemical indicator (liver function, renal function, fasting blood glucose). Exclusion criteria: (1) Lower extremity vascular occlusion that could not be excluded by AS detection; (2) Abnormal ankle-brachial index (ABI) (ABI < 0.9 or > 1.4) or invalid baPWV value (baPWV = 0); (3) Missing key indicator (e.g., unmeasured fasting blood glucose, renal function, liver function, blood pressure, height, or weight); (4) RC < 0 mmol/L (considered a laboratory error). This study was approved by the Ethics Review Committee of Zigong Fourth People’s Hospital (Approval No.: 2024–019). Because this study used anonymized retrospective data, the requirement for re-signing the informed consent form was exempted upon approval by the Ethics Review Committee, and there was no risk of privacy disclosure of the participants. The flowchart of subject inclusion and exclusion criteria was shown in Fig. 1.

### Study methods

#### Biochemical indicator detection

All participants fasted for 8 h before venous blood collection. Venous blood samples were stored at 4 ℃ and immediately transported to the laboratory for analysis using an ADVIA 2400 automatic biochemistry analyzer (Siemens, Germany). The detected indicators included: Blood lipids: TC, TG, HDL-C, LDL-C; Liver function: alanine aminotransferase (ALT), aspartate aminotransferase (AST), AST/ALT ratio, total bilirubin (TBIL), direct bilirubin (DBIL), indirect bilirubin (IBIL), total protein (TP), albumin (ALB), globulin (GLB), and albumin/globulin ratio (ALG); Renal function: estimated glomerular filtration rate (eGFR), blood urea nitrogen (BUN), uric acid (UA), and creatinine (Cr); Blood glucose: fasting blood glucose (FBG).

#### Blood pressure measurement

Participants rested quietly for 5 min in a seated position before measurement. Medical staff used an Omron HEM-907 electronic sphygmomanometer (Omron, Japan) to measure systolic blood pressure (SBP) and diastolic blood pressure (DBP) at the right arm, aligned with heart level. Two consecutive measurements were taken, and the average value was used as the final blood pressure. A history of hypertension was defined as a self-reported previous diagnosis of hypertension or a current measurement showing a SBP ≥ 140 mmHg or a DBP ≥ 90 mmHg, coupled with two previous records from medical check-ups that also showed an SBP ≥ 140 mmHg or a DBP ≥ 90 mmHg.

#### baPWV measurement

Participants were measured using an Omron BP-203 RPE Ⅲ detector (Omron, Japan), with cuffs placed on their upper arms and ankles. A heart sound detector was positioned at the left sternal edge of the participants, and electrocardiogram electrodes were attached to both their wrists. The device automatically recorded the participants’ baPWV (left and right sides) and ankle-brachial index (ABI). The higher value of the left and right baPWV of each subject was used, and AS was defined as baPWV ≥ 1800 cm/s in accordance with Chinese expert consensus [11].

#### Calculation of RC and Triglyceride-Glucose (TyG) series indices

RC was calculated using the Martin-Hopkins method, which is recommended by the American Heart Association, with the formula: *RC=TC-HDL-C-LDL-C* [12]. TyG series indiceswere calculated using the method proposed by Simental-Mendia [13], including: $TyG=In\left(TG\:[mg/dL]\times\:FBG[mg/dL]/2\right)$; $TyG-BMI=TyG\times\:BMI\left(kg/m^2\right)$; $TyG-WC=TyG\times\:waist\:circumference\left(WC,cm\right)$;$TyG-WHtR=TyG\times\:\left(WC[cm]\div\:height[cm]\right)$.

### Statistical methods

All statistical analyses were conducted with R software (version 4.5.1). Descriptive statistics: Normally distributed continuous variables were presented as mean ± standard deviation (*x* ± *s*), and comparisons between groups were conducted using the independent samples t-test. Non-normally distributed continuous variables were expressed as median [interquartile range] (*M [Q1, Q3]*), and comparisons were made using the Wilcoxon rank-sum test. Categorical variables were expressed as counts (percentages), and comparisons were performed using the χ^2^ test.

Multivariable logistic regression analysis was used to explore the independent and interactive associations of hypertension and elevated RC with AS, and three regression models with stepwise adjustment for confounding factors were constructed as follows: Model 1: No confounding factors adjusted; Model 2: Adjusted for age and gender; Model 3: Adjusted for age, gender, BMI, smoking history, drinking history, diabetes history, ALT, AST, TBIL, DBIL, ALB, GLB, eGFR, BUN, UA, Cr and FBG. Because RC is mathematically derived from TC, LDL-C, and HDL-C and is highly correlated with lipid-related variables, these components were not included in the fully adjusted model to avoid overadjustment and potential multicollinearity. For different analytical objectives, separate regression models were constructed, including independent-effect models, joint-exposure models, and additive-interaction models. Although the same covariates were adjusted in Models 1–3, the exposure variables were parameterized differently across analyses; therefore, the corresponding odds ratios are not directly comparable. The relative excess risk due to interaction (RERI), attributable proportion (AP), and synergy index (SI) were calculated to evaluate the additive interaction between hypertension and RC. Multicollinearity among covariates was evaluated using the variance inflation factor (VIF) and tolerance. A VIF > 10 was defined as indicative of significant multicollinearity.

Participants were also stratified by age (45–60 years, ≥ 60 years), gender, BMI (< 18.5, 18.5–23.9, 24–27.9, ≥ 28 kg/m^2^), smoking history, drinking history, diabetes history, and dyslipidemia history to explore subgroup differences in the interaction of hypertension and RC. Restricted cubic spline (RCS) model was used to analyze the non-linear relationship between SBP, DBP, RC, and AS risk.

## Results

### Basic characteristics of the study population

A total of 6,886 participants were included, with 4,702 (68.3%) in the AS group and 2,184 (31.7%) in the normal group. For baseline characteristics, the AS group showed significantly higher age, weight, waist circumference, SBP, and DBP (*P* < 0.001) and lower height (*P* < 0.001) compared with the normal group. There was no significant difference in gender distribution between the two groups (*P* = 0.320).

Physical status and lifestyle: The proportions of overweight (41.77% vs. 36.58%), obesity (11.78% vs. 7.19%), and smoking (30.92% vs. 26.83%) were significantly higher in the AS group than in the normal group (*P* < 0.001). No significant difference in drinking rate was observed (*P* = 0.064). Regarding comorbidities: The prevalences of hypertension (32.67% vs. 13.28%), diabetes (14.42% vs. 4.72%), and dyslipidemia (68.63% vs. 60.21%) in the AS group were significantly higher than those in the normal group (*P* < 0.001). Detailed basic characteristics are presented in Table 1.

**Table 1:** The basic characteristics of the study population

Variable	Total Population (*n* = 6,886)	Normal Group (*n* = 2,184)	AS Group (*n* = 4,702)	*P* Value
Age (years)	59.78 ± 10.03	58.93 ± 9.17	60.17 ± 10.39	< 0.001
Waist circumference (cm)	82.82 ± 8.82	81.05 ± 8.84	83.64 ± 8.69	< 0.001
SBP (mmHg)	128.23 ± 19.03	116.85 ± 15.05	133.51 ± 18.36	< 0.001
DBP (mmHg)	74.97 ± 11.72	69.61 ± 9.73	77.45 ± 11.74	< 0.001
Gender, *n* (%)				0.320
Male	4,235 (61.50)	1,324 (60.62)	2,911 (61.91)	
Female	2,651 (38.50)	860 (39.38)	1,791 (38.09)	
BMI (kg/m^2^), *n* (%)				< 0.001
< 18.5	192 (2.79)	79 (3.62)	113 (2.40)	
18.5–23.9	3,220 (46.76)	1,149 (52.61)	2,071 (44.05)	
24–27.9	2,763 (40.12)	799 (36.58)	1,964 (41.77)	
≥ 28	711 (10.33)	157 (7.19)	554 (11.78)	
Smoking, *n* (%)				< 0.001
No	4,846 (70.37)	1,598 (73.17)	3,248 (69.08)	
Yes	2,040 (29.63)	586 (26.83)	1,454 (30.92)	
Drinking, *n* (%)				0.064
No	4,008 (58.21)	1,307 (59.84)	2,701 (57.44)	
Yes	2,878 (41.79)	877 (40.16)	2,001 (42.56)	
Hypertension, *n* (%)				< 0.001
No	5,060 (73.48)	1,894 (86.72)	3,166 (67.33)	
Yes	1,826 (26.52)	290 (13.28)	1,536 (32.67)	
Diabetes, *n* (%)				< 0.001
No	6,105 (88.66)	2,081 (95.28)	4,024 (85.58)	
Yes	781 (11.34)	103 (4.72)	678 (14.42)	
Dyslipidemia, *n* (%)				< 0.001
No	2,344 (34.04)	869 (39.79)	1,475 (31.37)	
Yes	4,542 (65.96)	1,315 (60.21)	3,227 (68.63)	

Statistical methods: Normally distributed continuous variables (mean ± SD) by independent samples t-test; non-normally distributed variables (*M [Q1, Q3]*) by Wilcoxon rank-sum test; categorical variables (n, %) by *χ*^*2*^ test

*Abbreviations: AS* Arterial stiffness, *BMI* Body mass index, *SBP* Systolic blood pressure, *DBP* Diastolic blood pressure

### Independent effects of hypertension and RC on AS

Hypertension demonstrated a strong association with AS across all models. In the unadjusted model, the OR was 3.17 (95% CI: 2.76–3.64); following adjustment for age and gender, the OR slightly increased to 3.21 (95% CI: 2.78–3.70); in the fully adjusted model, the OR remained significant at 2.77 (95% CI: 2.38–3.22).

For RC, a significant association with AS was also observed across all models. The unadjusted OR was 1.70 (95% CI: 1.51–1.91); after adjustment for age and gender, the OR was 1.73 (95% CI: 1.51–1.95); following full confounder adjustment, the OR moderately decreased to 1.29 (95% CI: 1.13–1.49) while remaining statistically significant.

**Fig. 2:**
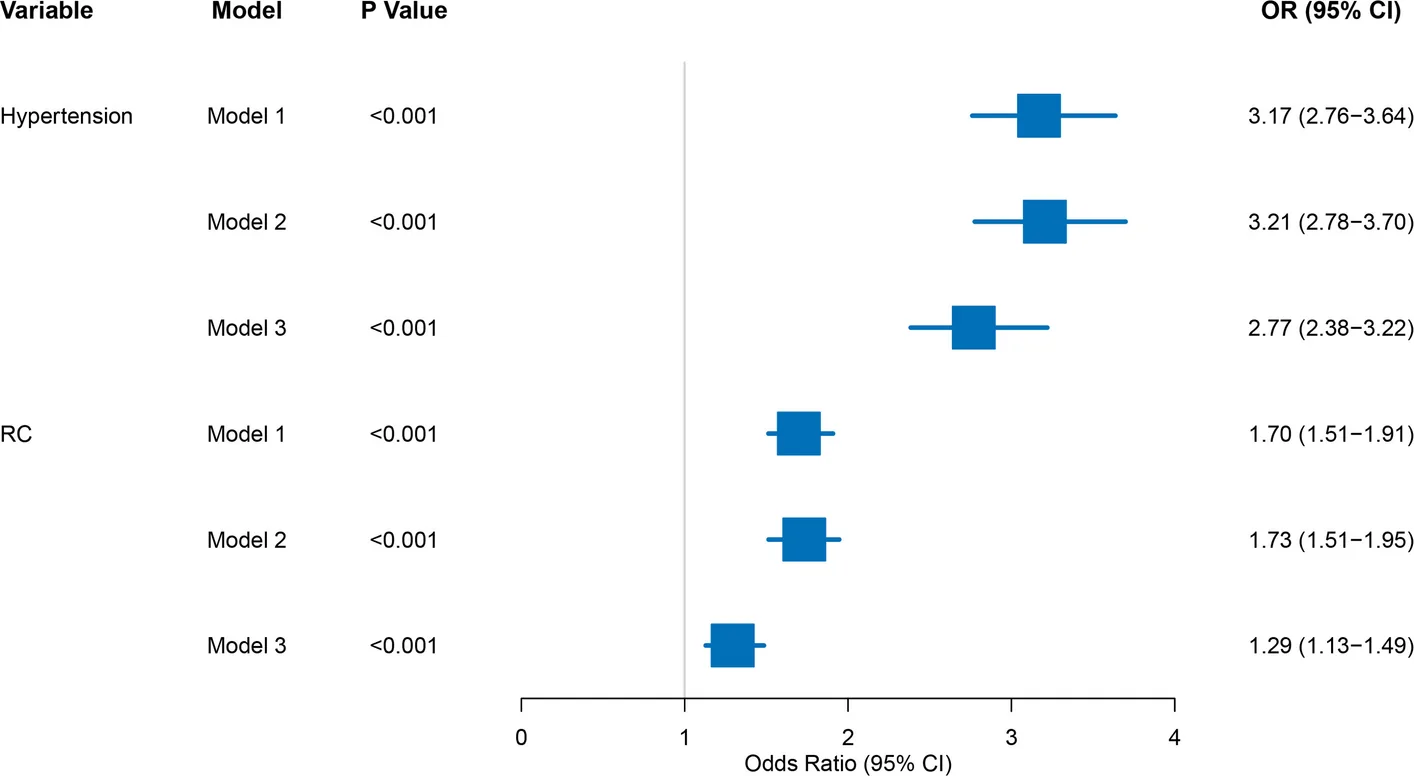
The independent effects of hypertension and elevated RC on AS. Note: Model 1: No confounding factors adjusted; Model 2: Adjusted for age and gender; Model 3: Adjusted for age, gender, BMI, smoking history, drinking history, diabetes history, ALT, AST, TBIL, DBIL, ALB, GLB, eGFR, BUN, UA, Cr and FBG

Notably, hypertension showed consistently higher OR values than RC across all three models, suggesting a stronger association with AS. Detailed results are displayed in Fig. 2.

### Joint effect of hypertension and RC on AS

Participants were divided into four subgroups: low RC without hypertension (Group 1, *n* = 3,540), high RC without hypertension (Group 2, *n* = 1,520), low RC with hypertension (Group 3, *n* = 1,225), and high RC with hypertension (Group 4, *n* = 601). The joint effect of hypertension and RC on AS was examined using three adjusted models.

Taking Model 3 (the fully adjusted model) as the reference: Compared with the low RC without hypertension group (reference, OR = 1), the high RC without hypertension group had an adjusted OR of 1.32 (95% CI: 1.14–1.53); the low RC with hypertension group had an adjusted OR of 2.83 (95% CI: 2.38–3.36); the high RC with hypertension group had the highest adjusted OR of 3.50 (95% CI: 2.66–4.61).

**Fig. 3:**
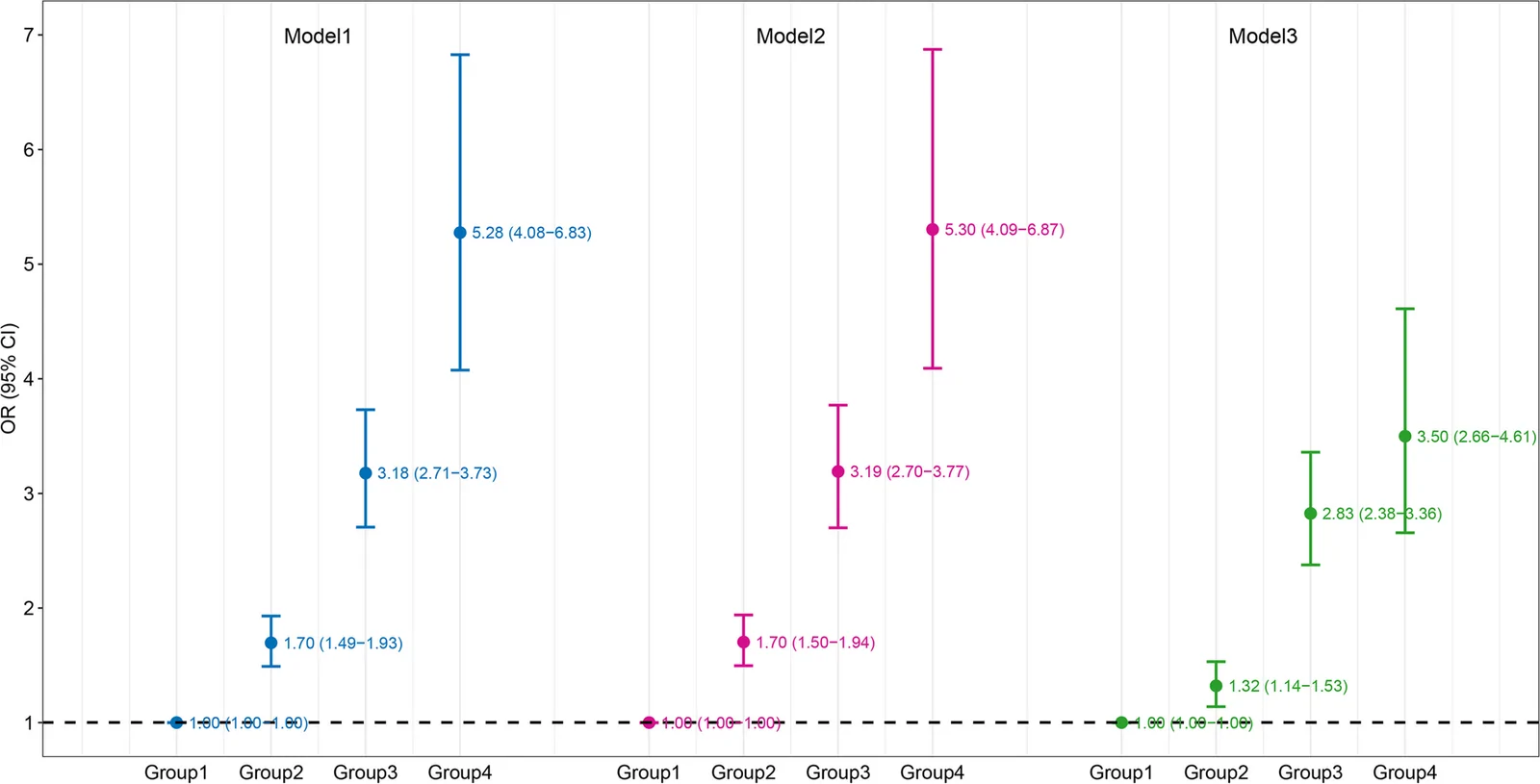
The joint effect of hypertension and elevated RC on AS. Note: Model 1: No confounding factors adjusted; Model 2: Adjusted for age and gender; Model 3: Adjusted for age, gender, BMI, smoking history, drinking history, diabetes history, ALT, AST, TBIL, DBIL, ALB, GLB, eGFR, BUN, UA, Cr and FBG

Across all three models, the trend in AS risk was consistent: low RC without hypertension < high RC without hypertension < low RC with hypertension < high RC with hypertension. With incremental adjustment for confounding factors, the OR values of each subgroup gradually stabilized, suggesting the consistency of the observed joint effect across different adjustment models. The forest plot of the joint effect is presented in Fig. 3.

### Additive interaction between hypertension and RC on AS

The additive interaction model was constructed to quantify the additive interaction between hypertension and RC on AS. After adjusting for multiple confounding factors, the results were as follows:

Independent effects: The independent effect of hypertension ranged from 2.83 (Model 3) to 3.18 (Model 1), while that of elevated RC ranged from 1.32 (Model 3) to 1.70 (Model 1). These results were consistent with those in Sect. " Independent effects of hypertension and RC on AS", confirming that both are independent risk factors for AS.

Additive interaction: The combined effect of hypertension and RC was stronger than the sum of their independent effects, with the adjusted OR increasing from 3.19 (Model 3) to 5.28 (Model 1).

Interaction indices: RERI: 1.16–1.40, indicating excess risk from the additive interaction; AP: 0.15–0.27 (95% CI excluding 0), meaning 15%−27% of AS risk in co-exposed participants was attributable to the interaction; SI ranged from 1.16 to 1.49 (95% CI excluding 1), suggesting the presence of an additive interaction between hypertension and elevated RC. However, the magnitude of the interaction was attenuated after full adjustment for confounding factors. Detailed results are presented in Table 2.

**Table 2:** Additive interaction between hypertension and RC on AS

Interaction Index	Model 1	Model 2	Model 3
Independent effect of hypertension (OR, 95% CI)	3.18 (2.71, 3.73)	3.19 (2.70, 3.77)	2.83 (2.38, 3.77)
Independent effect of RC (OR, 95% CI)	1.70 (1.49, 1.93)	1.7 (1.50, 1.94)	1.32 (1.14, 1.54)
Additive interaction effect (OR, 95% CI)	5.28 (4.08, 6.83)	5.30 (4.09, 6.87)	3.51 (2.66, 4.62)
RERI (95% CI)	1.40 (0.1, 2.81)	1.41 (0.12, 2.82)	1.21 (0.13, 2.23)
AP (95% CI)	0.27 (0.06, 0.47)	0.27 (0.06, 0.47)	0.15 (0.09, 0.31)
SI (95% CI)	1.49 (1.04, 2.12)	1.49 (1.04, 2.12)	1.16 (1.01, 1.77)

Model 1–3 are the same as those in Fig. 2

### Stratified analysis of the additive interaction

Stratified analysis was conducted to examine subgroup differences in the interaction between hypertension and RC on AS.

The interaction was more pronounced in females (*P* = 0.025), participants with normal BMI (*P* = 0.001), non-smokers (*P* = 0.028), and non-drinkers (*P* = 0.041). No significant inter-group differences were observed for age, diabetes, or dyslipidemia stratification (all *P* > 0.05). Detailed results are presented in Table 3.

**Table 3:** Stratified analysis of the interaction of hypertension and RC on AS

Stratification Factor	Group 1	Group 2	Group 3	Group 4	*P* Value
Age (years)					0.793
45–60	reference	1.76 (1.50–2.07)	3.39 (2.59–4.43)	5.09 (3.54–7.33)	
≥ 60	reference	1.58 (1.27–1.97)	3.01 (2.44–3.71)	5.38 (3.72–7.78)	
Gender					0.025
Male	reference	1.86 (1.58–2.20)	2.90 (2.40–3.52)	4.92 (3.63–6.67)	
Female	reference	1.46 (1.19–1.80)	4.10 (3.02–5.56)	6.43 (3.91–10.55)	
BMI (kg/m^2^)					0.001
< 18.5	reference	1.54 (0.64–3.72)	4.12 (1.33–12.72)	2.60 (0.26–25.61)	
18.5–23.9	reference	1.56 (1.29–1.88)	4.94 (3.73–6.54)	5.85 (3.55–9.64)	
24–27.9	reference	1.68 (1.37–2.06)	2.39 (1.88–3.03)	4.49 (3.15–6.40)	
≥ 28	reference	1.52 (0.96–2.43)	1.57 (1.00–2.45)	3.83 (1.97–7.45)	
Smoking History					0.028
No	reference	1.47 (1.26–1.72)	3.12 (2.59–3.75)	5.93 (4.22–8.34)	
Yes	reference	2.12 (1.68–2.67)	3.35 (2.42–4.63)	4.38 (2.93–6.54)	
Drinking History					0.041
No	reference	1.63 (1.36–1.95)	3.55 (2.89–4.37)	7.01 (4.77–10.30)	
Yes	reference	1.71 (1.41–2.07)	2.64 (2.04–3.42)	3.91 (2.75–5.56)	
Diabetes History					0.24
No	reference	1.62 (1.41–1.85)	3.15 (2.65–3.73)	4.59 (3.47–6.07)	
Yes	reference	1.56 (0.92–2.63)	1.75 (1.02–3.01)	4.07 (1.93–8.59)	
Dyslipidemia History					0.826
No	reference	1.99 (1.04–3.80)	3.08 (2.45–3.86)	6.86 (0.87–54.27)	
Yes	reference	1.55 (1.34–1.80)	3.28 (2.61–4.12)	4.82 (3.69–6.30)	

### Non-linear association between blood pressure, RC, and AS

To further characterize the potential non-linear association between blood pressure and AS risk, an RCS model was constructed after adjusting for confounding factors including age, gender, BMI, smoking history, drinking history, diabetes, dyslipidemia, liver function, renal function, and fasting blood glucose. Three internal nodes were set in the model, which were determined based on the 10th, 50th, and 90th percentiles of the distribution of SBP, DBP, and RC in the study population.

The RCS model revealed a significant non-linear association between blood pressure and AS risk (P for non-linearity < 0.05). Within the study population, the risk of AS appeared to increase more rapidly around SBP 127 mmHg and DBP 84 mmHg. However, these values should be interpreted as exploratory inflection points derived from the observed data distribution rather than definitive biological or clinical thresholds. Therefore, they should not be regarded as clinical intervention targets and require validation in prospective studies.

**Fig. 4:**
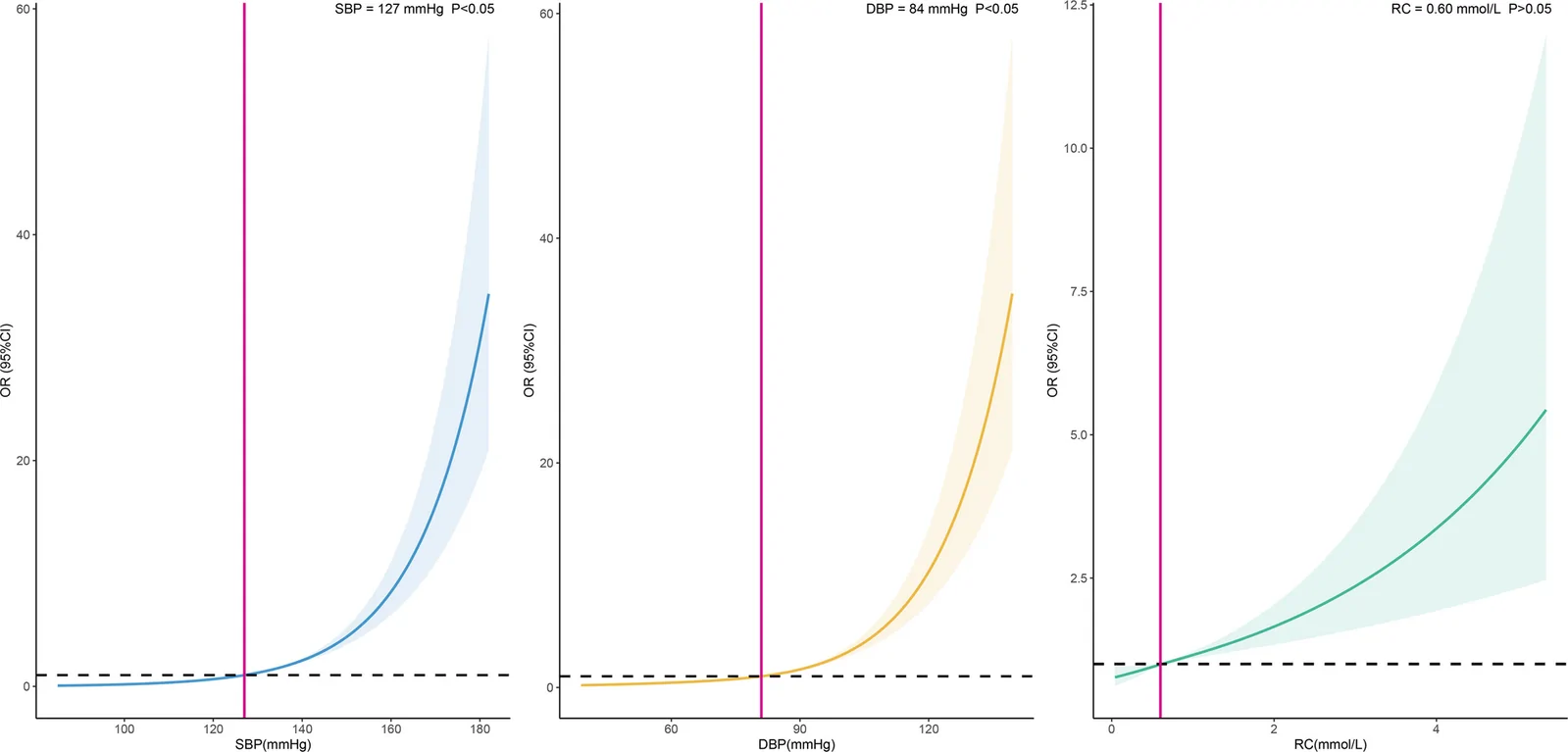
Restricted cubic spline curves of SBP, DBP, and RC with AS risk. (Note: The x-axis represents the actual measured values of SBP (mmHg), DBP (mmHg), and RC (mmol/L), respectively; the y-axis represents the adjusted OR for AS risk, with the median value of each exposure variable as the reference (OR = 1); the shaded area represents the 95% confidence interval (95% CI) of the OR; the vertical line indicates the approximate inflection points observed in the RCS curves, where the risk of AS appeared to increase more rapidly within the study population

In contrast, no non-linear association was found between RC and AS risk (*P*_for non-linearr_ > 0.05), indicating that AS risk increased linearly with rising RC levels, with no apparent threshold effect.. Detailed results are shown in Fig. 4.

## Discussion

Building upon previous evidence regarding the associations of remnant cholesterol and hypertension with AS, the present study further evaluated whether these two factors jointly contribute to AS through an additive interaction framework. Our findings suggest that hypertension and elevated RC are independently associated with AS and may exert a modest additive interaction, providing complementary evidence for integrated cardiovascular risk assessment in middle-aged and elderly populations. The main findings are as follows: (1) Hypertension and RC are independent risk factors for AS, with hypertension exhibiting a stronger association; (2) Hypertension and RC demonstrated a additive interaction on AS, with the highest risk observed in participants with both high RC and hypertension; (3) RCS analyses revealed a significant non-linear association between blood pressure and AS risk, with apparent inflection points around SBP 127 mmHg and DBP 84 mmHg in the current study population.; (4) The interaction is more prominent in females and participants with normal BMI. These findings carry important clinical implications for the prevention and management of AS in middle-aged and elderly populations.

### Hypertension: a stronger independent risk factor for AS

Hypertension is a well-recognized independent risk factor for AS, and its association with AS has been extensively reported in previous research. A 2022 systematic review and meta-analysis reported that each 1 m/s increase in baPWV was associated with a 9% higher risk of hypertension (RR = 1.09, 95% CI: 1.05–1.12) [14]. Consistent with this, our study found that hypertension increased the risk of AS by approximately 2.75 times after adjusting for multiple confounding factors, which aligns with the results of a Beijing-based health management cohort (*n* = 8,960) [15]. Similarly, a study by Allison et al. involving African men identified SBP-second only to age-as a key determinant of baPWV magnitude, which independently explained 10% of its variance. Specifically, each 1 standard deviation increase in SBP (21.6 mmHg) was associated with a mean elevation of 102 cm/s in baPWV (*P* < 0.001) [16]. These results together confirm that the impact of blood pressure on AS is well-evidenced.

Notably, the RCS analysis suggested that the association between blood pressure and AS risk became steeper around SBP 127 mmHg and DBP 84 mmHg within the present study population. Pre-hypertension has been reported to accelerate arterial elastic decline [17]. Consistent with these observations, our RCS analysis suggested a steeper increase in AS risk around SBP 127 mmHg and DBP 84 mmHg within the present study population. However, these values should be interpreted as exploratory inflection points rather than clinical thresholds, as they may be influenced by the underlying distribution of blood pressure in the study population. Given the cross-sectional design of this study, the observed associations cannot establish causality or support specific intervention targets. Further prospective studies are required to determine the clinical significance of these findings [18].

### Elevated RC: an independent risk factor for AS

RC, as a byproduct of triglyceride-rich lipoprotein metabolism, has gained attention as a marker of residual cardiovascular risk. Wadström et al. reported that participants with RC ≥ 1.5 mmol/L had a 2.4-fold higher risk of ischemic heart disease compared with those with RC < 0.5 mmol/L (HR = 2.4, 95% CI: 1.9–2.9) [19]. A Chinese community-based study also found a significant association between RC and baPWV [8].

Compared with the low-RC group, individuals in the high-RC group exhibited 21%−70% higher odds of AS across different adjustment models (OR 1.21–1.70), a stronger effect size than that reported in European populations. This discrepancy may be due to the lower baseline RC level in our cohort (median 0.64 mmol/L) compared with that of Wadström’s study populations, highlighting the need for population-specific risk assessment [20].

Importantly, we did not find non-linear association between RC and AS, suggesting that no apparent “safe threshold” for RC in middle-aged and elderly adults. This implies that even modest increases in RC may contribute to AS, underscoring the need for comprehensive lipid management that includes RC monitoring, in addition to LDL-C.

### Additive interaction between hypertension and RC

Recent studies have explored the relationship between RC, blood pressure, and AS from different analytical perspectives. For example, blood pressure has been reported as a potential mediator linking RC and AS. In contrast, the present study focused on additive interaction rather than mediation.

The present study observed an additive interaction between hypertension and elevated RC. Although the magnitude of the interaction was attenuated after adjustment for potential confounding factors (SI decreased from 1.49 to 1.16), the fully adjusted model still suggested a modest interaction between the two exposures.

The potential mechanisms underlying this effect may include: (1) Vascular endothelial dysfunction: Hypertension-induced mechanical shear stress increases endothelial permeability, facilitating the deposition of RC-rich lipoprotein remnants into the subendothelial space [21, 22]. This process accelerates the formation of atherosclerotic plaques and promotes AS. (2) Inflammatory activation: RC can activate the TLR4/NF-κB signaling pathway, triggering a pro-inflammatory response [23, 24]. Hypertension further exacerbates endothelial inflammation by increasing reactive oxygen species production, creating a “vicious cycle” that accelerates AS progression. (3) Vascular smooth muscle cell (VSMC) dysfunction: Both hypertension and RC can induce VSMC phenotypic transformation from a contractile to a synthetic phenotype, promoting collagen deposition and arterial wall fibrosis [25, 26].

Beyond the aforementioned mechanisms, AS pathogenesis involves more complex interactions. The cross-talk between metabolic risk factors and kidney function is critical: obesity and metabolic syndrome perturb renal function via altered adipokines, oxidative stress, and inflammation, while renal damage (e.g., microalbuminuria) exacerbates AS risk, forming a vicious cycle [27]. Similarly, adiposity interacts with extra-renal mineralocorticoid receptor (MR), whose abnormal activation promotes hypertension and obesity, with high aldosterone levels further disrupting metabolic-vascular homeostasis and exacerbating AS [28]. AS is also intricately linked to multiple organ damage, as described by the systemic hemodynamic atherothrombotic syndrome (SHATS), which involves a synergistic cycle of hemodynamic stress (including BPV) and vascular disease that mutually amplifies AS and BP elevation [29]. Additionally, arterial remodelling—particularly the dilated hypertrophic carotid phenotype (dh-CGP)—independently accelerates arterial stiffening and AS progression, acting as a key mediator of hypertension- and dyslipidemia-induced arterial damage [30]. It should also be noted that the magnitude of the additive interaction was sensitive to covariate adjustment. The attenuation observed in the fully adjusted model suggests that part of the joint effect may be explained by shared metabolic and clinical risk factors.

### Subgroup differences: implications for precision medicine

Our stratified analysis revealed that the interaction of hypertension and RC was more prominent in females and participants with normal BMI.

Gender differences: Females in the high RC with hypertension group had a 6.43-fold higher AS risk compared with 4.92-fold in males. This aligns with a Norwegian study that found a stronger association between midlife blood pressure and late-life AS in females (OR = 4.62 for hypertension vs. 2.78 for elevated blood pressure) [31]. The underlying reason may be the loss of estrogen protection after menopause, which increases vascular sensitivity to lipid and blood pressure abnormalities [32]. Notably, previous studies have demonstrated sex differences in salt-sensitivity of blood pressure and the impact of endothelial dysfunction, which may further contribute to the more prominent interaction observed in females in our study [33]. Salt sensitivity is more prevalent in females, especially postmenopausal women, and is closely associated with endothelial dysfunction, which can amplify the adverse effects of hypertension and elevated RC on AS [34].

BMI differences: The relatively stronger interaction was observed in participants with normal BMI (18.5–23.9 kg/m^2^), with an OR of 5.85 (95% CI: 3.72–7.78). While mainstream perspectives highlight overweight/obesity as a primary driver of cardiovascular risk and underscore the relevance of “metabolically abnormal non-obesity” [35], this discrepancy stems from the specificity of the study population and outcome indicators specifically, the present study focuses on subclinical vascular damage (AS assessed by baPWV), whereas mainstream views are mostly derived from research targeting clinical cardiovascular endpoints such as myocardial infarction or stroke-rather than representing a contradiction between the two [36, 37]. Even in individuals with normal weight, the coexistence of hypertension and elevated RC can significantly increase AS risk, suggesting the necessity of metabolic screening for this population regardless of BMI. However, the study did not adequately explore sex differences in how obesity modulates the interaction of hypertension and RC on AS, which warrants further investigation in future research.

### Interpretation and generalizability of the observed interaction

It should also be noted that the magnitude of the observed additive interaction was sensitive to covariate adjustment. The attenuation observed in the fully adjusted model suggests that part of the joint effect may be explained by shared metabolic and clinical risk factors.

Another important issue that should be considered is the absence of information regarding antihypertensive and lipid-lowering medication use. Pharmacological treatment may substantially alter measured blood pressure and RC levels, resulting in potential exposure misclassification. For example, some participants with well-controlled hypertension or dyslipidemia may have been classified into the non-hypertensive or low-RC groups. Such non-differential misclassification could attenuate the observed associations and may lead to an underestimation of the additive interaction between hypertension and RC. However, because medication information was unavailable, the direction and magnitude of this potential bias cannot be quantified and should be addressed in future prospective studies. Therefore, the observed interaction should be interpreted conservatively.

Finally, another important issue that should be considered is the representativeness of the study population. Participants were recruited from a single health management center, and the prevalence of AS in this cohort was relatively high. In addition, the exclusion of individuals with lower-extremity vascular occlusion, abnormal ABI, and missing key clinical data may have further influenced the characteristics of the study population. Therefore, the observed magnitude of the additive interaction, the RCS-derived inflection points, and the subgroup-specific findings should be interpreted within the context of this selected population and may not be directly generalizable to the broader middle-aged and elderly Chinese population. Future multicenter prospective studies involving more diverse populations are needed to validate these findings.

### Limitations

This study has several limitations. First, participants were recruited from a single health management center. The high prevalence of AS (68.3%) may be attributed to healthy-user bias and selection bias, as health check-up attendees often have elevated cardiovascular risk. Additionally, we excluded subjects with missing metabolic and anthropometric data, or conditions including lower extremity vascular occlusion, zero baPWV and abnormal ABI. Second, information on antihypertensive and lipid-lowering medication use was not available. Because these medications may modify blood pressure, RC levels, and AS, residual confounding and exposure misclassification cannot be excluded. This may have influenced the estimated magnitude of the additive interaction observed in the present study. Third, we only analyzed baseline values and did not evaluate whether interventions targeting hypertension and RC could reverse or delay AS. Future randomized controlled trials are warranted to validate the efficacy of combined strategies. Fourth, although baPWV is widely used for assessing overall AS, it is not the gold standard for central aortic stiffness. Fifth, information on the use of antihypertensive and lipid-lowering medications was not systematically collected in this health check-up population, which may introduce residual confounding and affect the validity of the interaction estimates. Our Future study will use cfPWV to verify and extend our findings and endeavor to collect and adjust for medication data to improve result robustness.

## Conclusions

This study suggests that hypertension and elevated RC may jointly contribute to AS through a modest additive interaction in middle-aged and elderly Chinese populations. In addition, RCS analyses identified apparent inflection points around SBP 127 mmHg and DBP 84 mmHg within the study population. These findings suggest that the integrated assessment of hypertension and RC may provide complementary information for identifying individuals at increased risk of AS. However, the observed interaction and the RCS-derived inflection points should be interpreted cautiously and require validation in larger prospective multicenter studies.

## Supplementary Information

Supplementary Material 1.

## Data Availability

The data supporting the findings of this study are available from the Health Management Center of Zigong Fourth People’s Hospital. Due to privacy and ethical restrictions related to medical data, the data cannot be publicly shared. For access to the data, interested parties may send an email to zixian@gmail.com with a reasonable request.

## Abbreviations

AS: Arterial stiffness
CVD: Cardiovascular and cerebrovascular diseases
RC: Remnant cholesterol
baPWV: Brachial-ankle pulse wave velocity
SBP: Systolic blood pressure
DBP: Diastolic blood pressure
BMI: Body mass index
RCS: Restricted cubic spline
TC: Total cholesterol
TG: Triglycerides
HDL-C: High-density lipoprotein cholesterol
LDL-C: Low-density lipoprotein cholesterol
TyG: Triglyceride-glucose
TyG-BMI: Triglyceride-glucose-body mass index
TyG-WC: Triglyceride-glucose-waist circumference
TyG-WHtR: Triglyceride-glucose-waist-to-height ratio
ALT: Alanine aminotransferase
AST: Aspartate aminotransferase
TBIL: Total bilirubin
DBIL: Direct bilirubin
IBIL: Indirect bilirubin
ALB: Albumin
GLB: Globulin
eGFR: Estimated glomerular filtration rate
BUN: Blood urea nitrogen
UA: Uric acid
Cr: Creatinine
FBG: Fasting blood glucose
ABI: Ankle-brachial index
OR: Odds ratio
CI: Confidence interval
RERI: Relative excess risk due to interaction
AP: Attributable proportion
SI: Synergy index
DALYs: Disability-adjusted life years

## References

[1] Ajoolabady A, Pratico D, Lin L, et al. Inflammation in atherosclerosis: pathophysiology and mechanisms. Cell Death Dis. 2024;15(11):817. 39528464 10.1038/s41419-024-07166-8 PMC11555284

[2] Fan J, Watanabe T. Atherosclerosis: known and unknown. Pathol Int. 2022;72(3):151–60. 35076127 10.1111/pin.13202

[3] Herrington W, Lacey B, Sherliker P, et al. Epidemiology of atherosclerosis and the potential to reduce the global burden of atherothrombotic disease. Circ Res. 2016;118(4):535–46. 26892956 10.1161/CIRCRESAHA.115.307611

[4] Maruhashi T, Higashi Y. Current topic of vascular function in hypertension in 2023–2024. Hypertens Res. 2023;47(12):3310–7. 10.1038/s41440-024-01885-3 PMC11618089 39300292

[5] Ferrari AJ, Santomauro DF, Aali A, et al. Global incidence, prevalence, years lived with disability (YLDs), disability-adjusted life-years (DALYs), and healthy life expectancy (HALE) for 371 diseases and injuries in 204 countries and territories and 811 subnational locations, 1990–2021: a systematic analysis for the Global Burden of Disease Study 2021. The Lancet. 2024;403(10440):2133–61. 10.1016/S0140-6736(24)00757-8 PMC11122111 38642570

[6] Liu H-H, Guo Y-L, Zhu C-G, et al. Synergistic effect of the commonest residual risk factors, remnant cholesterol, lipoprotein (a), and inflammation, on prognosis of statin-treated patients with chronic coronary syndrome. J Transl Med. 2022;20(1):243. 35619146 10.1186/s12967-022-03448-x PMC9134647

[7] Fu J, Deng Y, Ma Y, et al. National and provincial-level prevalence and risk factors of carotid atherosclerosis in Chinese adults. JAMA Netw Open. 2024;7(1):e2351225. 38206625 10.1001/jamanetworkopen.2023.51225 PMC10784858

[8] Liu J, Fan F, Liu B, et al. Association between remnant cholesterol and arterial stiffness in a Chinese community-based population: a cross-sectional study. Front Cardiovasc Med. 2022;9:993097. 36440032 10.3389/fcvm.2022.993097 PMC9691684

[9] Wu W, Chen G, Wu K, et al. Cumulative exposure to high remnant-cholesterol concentrations increases the risk of cardiovascular disease in patients with hypertension: a prospective cohort study. Cardiovasc Diabetol. 2023;22(1):258. 37735420 10.1186/s12933-023-01984-4 PMC10515262

[10] Yu C, Wang T, Zhu L, et al. Impact of remnant cholesterol on arterial stiffness and mediating role of systolic blood pressure in Chinese hypertensive adults. BMC Public Health. 2025;25(1):3391. 41063077 10.1186/s12889-025-24635-7 PMC12506517

[11] Expert Consensus Drafting Group of the Subcommittee on Refractory Hypertension and Peripheral Arterial Disease, Chinese International Exchange and Promotion Association for Medical and Healthcare. Expert Consensus Drafting Group of the Subcommittee on Refractory Hypertension and Peripheral Arterial Disease, Chinese International Exchange and Promotion Association for Medical and Healthcare Chinese expert consensus on clinical application of simultaneous four-limb blood pressure and brachial-ankle pulse wave velocity measurement. Chin Circ J. 2020;35(6):521–8 (In Chinese).

[12] Varbo A, Benn M, Nordestgaard BG. Remnant cholesterol as a cause of ischemic heart disease: evidence, definition, measurement, atherogenicity, high risk patients, and present and future treatment. Pharmacol Ther. 2014;141(3):358–67. 24287311 10.1016/j.pharmthera.2013.11.008

[13] Simental-Mendía LE, Rodríguez-Morán M, Guerrero-Romero F. The product of fasting glucose and triglycerides as surrogate for identifying insulin resistance in apparently healthy subjects. Metab Syndr Relat Disord. 2008;6(4):299–304. 19067533 10.1089/met.2008.0034

[14] Saz-Lara A, Bruno RM, Cavero-Redondo I, et al. Association between arterial stiffness and blood pressure progression with incident hypertension: a systematic review and meta-analysis. Frontiers in Cardiovascular Medicine. 2022;9:798934. 35224042 10.3389/fcvm.2022.798934 PMC8873377

[15] Wu Z, Jiang Y, Zhu Q, et al. Combined evaluation of arterial stiffness and blood pressure promotes risk stratification of peripheral arterial disease. JACC Asia. 2023;3(2):287–97. 37181389 10.1016/j.jacasi.2023.02.001 PMC10167522

[16] Kuipers AL, Miljkovic I, Barinas-Mitchell E, Cvejkus R, Bunker CH, Wheeler VW, et al. Arterial stiffness and hypertension status in Afro-Caribbean men. J Hypertens. 2019;37(3):546–54. 30234778 10.1097/HJH.0000000000001909 PMC6355357

[17] Gedikli O, Kiris A, Ozturk S, et al. Effects of prehypertension on arterial stiffness and wave reflections. Clin Exp Hypertens. 2010;32(2):84–9. 20374190 10.3109/10641960902993103

[18] Ma S, Xie X, Yuan R, et al. Vascular aging and atherosclerosis: a perspective on aging. Aging Dis. 2024;16(1):33–48. 38502584 10.14336/AD.2024.0201-1 PMC11745439

[19] Wadström BN, Wulff AB, Pedersen KM, et al. Elevated remnant cholesterol increases the risk of peripheral artery disease, myocardial infarction, and ischaemic stroke: a cohort-based study. Eur Heart J. 2022;43(34):3258–69. 34661640 10.1093/eurheartj/ehab705

[20] Liu C, Dai M, Tian K, et al. Association of remnant cholesterol with CVD incidence: a general population cohort study in Southwest China. Front Cardiovasc Med. 2023;10:1286286. 38089771 10.3389/fcvm.2023.1286286 PMC10711629

[21] Zhou J, Li Y-S, Chien S. Shear stress–initiated signaling and its regulation of endothelial function. Arterioscler Thromb Vasc Biol. 2014;34(10):2191–8. 24876354 10.1161/ATVBAHA.114.303422 PMC4169328

[22] Chatterjee S. Endothelial mechanotransduction, redox signaling and the regulation of vascular inflammatory pathways. Front Physiol. 2018;9:524. 29930512 10.3389/fphys.2018.00524 PMC5999754

[23] Wilson SH, Caplice NM, Simari RD, et al. Activated nuclear factor-κB is present in the coronary vasculature in experimental hypercholesterolemia. Atherosclerosis. 2000;148(1):23–30. 10580167 10.1016/s0021-9150(99)00211-7

[24] Hajra L, Evans AI, Chen M, et al. The NF-κB signal transduction pathway in aortic endothelial cells is primed for activation in regions predisposed to atherosclerotic lesion formation. Proc Natl Acad Sci. 2000;97(16):9052–7. 10922059 10.1073/pnas.97.16.9052 PMC16820

[25] O’callaghan CJ, Williams B. Mechanical strain–induced extracellular matrix production by human vascular smooth muscle cells: Role of TGF-β1. Hypertension. 2000;36(3):319–24. 10988258 10.1161/01.hyp.36.3.319

[26] Wang M, Spinetti G, Monticone RE, et al. A local proinflammatory signalling loop facilitates adverse age-associated arterial remodeling. PLoS ONE. 2011;6(2):e16653. 21347430 10.1371/journal.pone.0016653 PMC3035650

[27] Tesauro M, Canale MP, Rodia G, et al. Metabolic syndrome, chronic kidney, and cardiovascular diseases: role of adipokines. Cardiol Res Pract. 2011;2011:653182. 21403882 10.4061/2011/653182 PMC3051177

[28] Feraco A, Marzolla V, Scuteri A, et al. Mineralocorticoid receptors in metabolic syndrome: from physiology to disease. Trends Endocrinol Metab. 2020;31(3):205–17. 31843490 10.1016/j.tem.2019.11.006

[29] Kario K, Chirinos JA, Townsend RR, et al. Systemic hemodynamic atherothrombotic syndrome (SHATS) - coupling vascular disease and blood pressure variability: proposed concept from pulse of Asia. Prog Cardiovasc Dis. 2020;63(1):22–32. 31810526 10.1016/j.pcad.2019.11.002

[30] AlGhatrif M, Lakatta EG, Morrell CH, et al. Dilated hypertrophic phenotype of the carotid artery is associated with accelerated age-associated central arterial stiffening. Geroscience. 2023;45(2):1001–13. 36520341 10.1007/s11357-022-00699-w PMC9886763

[31] Kringeland E, Midtbø H, Ohldieck AE, et al. Sex-specific associations between blood pressure in early midlife and arterial stiffness 27 years later: The Hordaland Health Study. Eur J Prevent Cardiol. 2025;33:379. 10.1093/eurjpc/zwaf379 40578832

[32] Yu Z, Yang H, Shou B, et al. Remnant cholesterol and the risk of carotid plaque in hypertension: results from a community-based screening among old adults in Hangzhou, China. Sci Rep. 2024;14(1):8407. 38600230 10.1038/s41598-024-58484-y PMC11006856

[33] Scuteri A, Stuehlinger MC, Cooke JP, Wright JG, Lakatta EG, Anderson DE, et al. Nitric oxide inhibition as a mechanism for blood pressure increase during salt loading in normotensive postmenopausal women. J Hypertens. 2003;21(7):1339–46. 12817182 10.1097/00004872-200307000-00023

[34] Masenga SK, Wandira N, Cattivelli-Murdoch G, et al. Salt sensitivity of blood pressure: mechanisms and sex-specific differences. Nat Rev Cardiol. 2025;22(9):611–28. 39984695 10.1038/s41569-025-01135-0 PMC13227966

[35] Wang CF, Xu HQ, Wu XY, et al. New understanding of metabolically healthy obesity and its research opportunities. Chin J Prevent Med. 2022;56(1):69–74 (In Chinese). 10.3760/cma.j.cn112150-20210205-00129 35092994

[36] Cao L, Zhu L, Zhang W, et al. Metabolic obesity phenotypes and risk of ischemic stroke: the Rural Chinese Cohort Study. Front Nutr. 2026;12:1749472. 41601886 10.3389/fnut.2025.1749472 PMC12833963

[37] Janota O, Mantovani M, Kwiendacz H, et al. Metabolically “extremely unhealthy” obese and non-obese people with diabetes and the risk of cardiovascular adverse events: the Silesia Diabetes - Heart Project. Cardiovasc Diabetol. 2024;23(1):326. 39227929 10.1186/s12933-024-02420-x PMC11373332

