# Supplementary material for: Interaction of hypertension and remnant cholesterol on arterial stiffness in Chinese middle-aged and elderly populations
Source: Lipids Health Dis. 2026 Jun 30;25:195. doi: 10.1186/s12944-026-03006-0 (PMC13579982; doi:10.1186/s12944-026-03006-0)
Supplement: Supplementary file 1 [file 12944_2026_3006_MOESM1_ESM.docx]

**Comparison of Biochemical Indicators Between Groups**

Blood lipids: The AS group had significantly higher levels of TC (5.08±1.02 vs. 5.02±0.94 mmol/L, *P*=0.020), TG (2.07±1.83 vs. 1.62±1.25 mmol/L, *P* < 0.001), and RC (0.77±0.61 vs. 0.64±0.42 mmol/L, *P* < 0.001), and a lower level of HDL-C (1.32±0.39 vs. 1.40±0.39 mmol/L, *P* < 0.001) compared with the normal group. No significant difference in LDL-C was observed (*P*=0.621).

Liver function: The AS group showed higher levels of ALT (25.52±16.84 vs. 23.15±15.81 mmol/L), AST (26.16±12.44 vs. 25.00±11.88 mmol/L), TP (74.95±4.14 vs. 73.94±3.96 g/L), ALB (45.71±2.64 vs. 45.46±2.61 μmol/L), and GLB (29.24±3.80 vs. 28.48±3.49 μmol/L) (all *P* < 0.001), and lower levels of AST/ALT (1.19±0.49 vs. 1.23±0.44), TBIL (16.67±6.04 vs. 17.50±6.35 μmol/L), DBIL (4.28±1.87 vs. 4.54±2.13 μmol/L), IBIL (12.39±4.49 vs. 12.96±4.60 μmol/L), and ALG (1.59±0.24 vs. 1.62±0.23) (all *P* < 0.001).

Renal function: The AS group had a higher UA level (372.29±89.16 vs. 358.51±84.04 mmol/L, *P* < 0.001) and a lower eGFR (93.39±14.60 vs. 94.20±13.09 mL/(min·1.73m²), *P*=0.027) than the normal group. No significant differences in BUN and Cr were found (*P*=0.414, *P*=0.503).

Blood glucose and TyG indices: The AS group had a higher FBG level (5.83±1.88 vs. 5.23±1.15 mmol/L, P < 0.001) and significantly higher TyG (7.33±0.70 vs. 7.05±0.58), TyG-BMI (179.37±32.50 vs. 167.38±28.93), TyG-WC (615.55±100.94 vs. 573.11±91.77), and TyG-WHtR (3.81±0.59 vs. 3.53±0.53) (all P < 0.001). Detailed biochemical indicators are presented in Appendix 1.

Appendix 1 Comparison of biochemical indicators between the normal and AS group

| Variable | Total Population (n=6,886) | Normal Group (n=2,184) | AS Group (n=4,702) | *P* Value |
| --- | --- | --- | --- | --- |
| TC (mmol/L) | 5.06±1.00 | 5.02±0.94 | 5.08±1.02 | 0.020 |
| TG (mmol/L) | 1.93±1.68 | 1.62±1.25 | 2.07±1.83 | <0.001 |
| LDL-C (mmol/L) | 2.99±0.83 | 2.99±0.79 | 3.00±0.84 | 0.621 |
| HDL-C (mmol/L) | 1.34±0.39 | 1.40±0.39 | 1.32±0.39 | <0.001 |
| RC (mmol/L) | 0.73±0.56 | 0.64±0.42 | 0.77±0.61 | <0.001 |
| ALT (mmol/L) | 24.77±16.56 | 23.15±15.81 | 25.52±16.84 | <0.001 |
| AST (mmol/L) | 25.79±12.27 | 25.00±11.88 | 26.16±12.44 | <0.001 |
| AST/ALT | 1.21±0.48 | 1.23±0.44 | 1.19±0.49 | <0.001 |
| TBIL (μmol/L) | 16.93±6.15 | 17.50±6.35 | 16.67±6.04 | <0.001 |
| DBIL (μmol/L) | 4.37±1.96 | 4.54±2.13 | 4.28±1.87 | <0.001 |
| IBIL (μmol/L) | 12.57±4.53 | 12.96±4.60 | 12.39±4.49 | <0.001 |
| TP (g/L) | 74.63±4.11 | 73.94±3.96 | 74.95±4.14 | <0.001 |
| ALB (μmol/L) | 45.63±2.63 | 45.46±2.61 | 45.71±2.64 | <0.001 |
| GLB (μmol/L) | 29.00±3.72 | 28.48±3.49 | 29.24±3.80 | <0.001 |
| ALG | 1.60±0.24 | 1.62±0.23 | 1.59±0.24 | <0.001 |
| eGFR (mL/(min·1.73m²)) | 93.65±14.14 | 94.20±13.09 | 93.39±14.60 | 0.027 |
| BUN (mmol/L) | 5.41±1.48 | 5.43±1.43 | 5.40±1.51 | 0.414 |
| UA (mmol/L) | 367.92±87.79 | 358.51±84.04 | 372.29±89.16 | <0.001 |
| Cr (mmol/L) | 69.67±18.94 | 69.45±17.39 | 69.78±19.62 | 0.503 |
| FBG (mmol/L) | 5.64±1.71 | 5.23±1.15 | 5.83±1.88 | <0.001 |
| TyG | 7.24±0.68 | 7.05±0.58 | 7.33±0.70 | <0.001 |
| TyG-BMI | 175.57±31.90 | 167.38±28.93 | 179.37±32.50 | <0.001 |
| TyG-WC | 602.09±100.09 | 573.11±91.77 | 615.55±100.94 | <0.001 |
| TyG-WHtR | 3.72±0.59 | 3.53±0.53 | 3.81±0.59 | <0.001 |

Abbreviations:TC=Total cholesterol; TG=Triglycerides; LDL-C=Low-density lipoprotein cholesterol; HDL-C=High-density lipoprotein cholesterol; RC=Remnant cholesterol; ALT=Alanine aminotransferase; AST=Aspartate aminotransferase; TBIL=Total bilirubin; DBIL=Direct bilirubin; IBIL=Indirect bilirubin; TP=Total protein; ALB=Albumin; GLB=Globulin; ALG=Albumin/globulin ratio; eGFR=Estimated glomerular filtration rate; BUN=Blood urea nitrogen; UA=Uric acid; Cr=Creatinine; FBG=Fasting blood glucose; TyG=Triglyceride-glucose; TyG-BMI=Triglyceride-glucose-body mass index; TyG-WC=Triglyceride-glucose-waist circumference; TyG-WHtR=Triglyceride-glucose-waist-to-height ratio; AS=Arterial stiffness.

**Multicollinearity Test of the Multivariable logistic regression model**

To examine the presence of multicollinearity among independent variables in the adjusted multivariable regression model, we performed a multicollinearity test on Model 3 (adjusted for age, gender, BMI, smoking history, drinking history, history of diabetes, history of dyslipidemia, ALT, AST, TBIL, DBIL, ALB, GLB, eGFR, BUN, UA, Cr, and FBG). The results showed that the variance inflation factor (VIF) of all adjusted variables was less than 10, with detailed values presented as follows:

Appendix 2 Multicollinearity Test of the Multivariable logistic regression model

| VIF of adjusted variable | Independent effct of Hypertension | Independent effct of RC |
| --- | --- | --- |
| Gender | 3.628 | 3.628 |
| Age | 3.686 | 3.610 |
| BMI | 1.210 | 1.183 |
| Smoking | 1.728 | 1.746 |
| Drinking | 2.164 | 2.164 |
| Diabetes | 2.711 | 2.709 |
| Dyslipidemia | 1.322 | 1.436 |
| ALT | 2.745 | 2.744 |
| AST | 2.676 | 2.673 |
| TBIL | 3.422 | 3.579 |
| DBIL | 3.025 | 3.140 |
| ALB | 1.203 | 1.514 |
| GLB | 1.233 | 1.472 |
| GFR | 9.563 | 9.560 |
| BUN | 1.296 | 1.297 |
| UA | 1.536 | 1.545 |
| Cr | 8.232 | 8.232 |
| FBG | 2.766 | 2.772 |
